# Linezolid dose adjustment according to therapeutic drug monitoring helps reach the goal concentration in severe patients, and the oldest seniors benefit more

**DOI:** 10.1186/s12879-023-08831-7

**Published:** 2023-11-29

**Authors:** Ying Xu, Xilan Yang, Pei Liang, Chen Qu

**Affiliations:** 1 Department of Intensive Care Unit, Nanjing Drum Tower Hospital, The Affiliated Hospital of Nanjing University Medical School, Nanjing, 210000 China; 2 Department of General Practice, The Fourth Affiliated Hospital of Nanjing Medical University, Nanjing, 210000 China; 3 Department of Pharmacy, Nanjing Drum Tower Hospital, The Affiliated Hospital of Nanjing University Medical School, Nanjing, 210000 China; 4 Geriatric Medicine Department, The Second Affiliated Hospital of Nanjing Medical University, Jiangjiayuan 121#, Nanjing, 210000 China

**Keywords:** Linezolid, Therapeutic drug monitoring, Trough concentration, The elderly

## Abstract

**Background:**

The elderly with severe infection increased dramatically in intensive care unit (ICU). Proper antimicrobial therapy help improve the prognosis. Linezolid, as an antimicrobial drug, is commonly utilized to treat patients infected with methicillin-resistant S. aureus and vancomycin-resistant enterococci. Clinical evidence suggests elderly patients prone to linezolid overexposure. Here, we describe the results of three years’ linezolid adjustment experiences according to therapeutic drug monitoring (TDM), especially in the oldest old.

**Methods:**

Linezolid therapeutic drug monitoring data were collected between January 2020 and November 2022 from patients who were admitted to ICU and treated with linezolid. All the patients started with a dosage of 600 mg, twice daily. The first TMD was carried out ten minutes before the seventh administration. The dosage adjustment was determined by the doctor according to the first TMD and patients' condition, and the repeated TDM was conducted as required. The dosage adjustment in different age group was recorded. Laboratory data were compared between the old and the oldest old. The high mortality risk of the oldest old was also explored.

**Results:**

Data of 556 linezolid TDM from 330 patients were collected. Among which, 31.6%, 54.8%, and 75% of patients had supra-therapeutic linezolid trough concentrations at the first TDM assessment in different age group, leading to the dosage adjustment rate of 31.0%, 40.3%, 68.8% respectively. The linezolid dosage adjustments according to TDM help to reach therapeutic concentration. The oldest old was in high risk of linezolid overexposure with lowercreatinine clearance. The norepinephrine maximum dosage but not linezolid C_min_ was associated with 28-day mortality in the oldest old.

**Conclusions:**

Elderly patients with linezolid conventional 600 mg twice-daily dose might be at a high risk of overexposure, especially in the oldest old. The linezolid dosage adjustments according to TDM help reach the therapeutic concentration. The high mortality of the oldest old was not related with initial linezolid overexposure.

## Introduction

Severe infection is common and frequently fatal in the elderly in intensive care unit (ICU) [1]. Mortality was increased with age, from 10% in children to 38.4% in those > 85 years old [2]. Proper antimicrobial therapy help improve the prognosis [3]. The methicillin-resistant S. aureus and vancomycin-resistant enterococci are considered the most common gram-positive bacteria found in ICU, and linezolid, as an antimicrobial drug, is commonly utilized to treat patients [4].

Various antibiotics vary from different pharmacodynamic indexes. Vancomycin, for example, the optimal therapeutic pharmacokynetic/pharmacodynamic (PK/PD) indices is area under the 24 h concentration–time curve / the minimum inhibitory concentration (AUC/MIC) > 400, approximately trough concentration (C_min_) 15–20 mg/L [5, 6]. Linezolid is a kind of time-dependent antibiotic, the AUC/MIC > 80 and the percentage of time that the plasma concentrations surpass the MIC (T > MIC = 85–100%) are the optimal PK/PD indices. Linezolid C_min_ is commonly used in practice instead. After the initial linezolid treatment, the C_min_ is assumed to be maintained between 2 to 7 μg/mL [7]. In general, linezolid is administered at a dose of 600 mg twice daily via oral and/or intravenous infusion. Dosing adjustment is considered unnecessary in elderly patients given on the drug’s label sheet. However, some evidences show significant association between patients’ age and linezolid exposure [8–10].

Compared with younger patients, the old and the very old patients have linezolid C_min_ three-fold higher when treated with the conventional 600-mg twice daily dose [10]. A prospective pilot study revealed the fact that patients aged > 70 years had drug C_min_ exceeding 8 mg/L treated with the conventional dose [10]. Increasing evidence demonstrated that exceeding the upper therapeutic safety threshold might lead to linezolid-related adverse events, such as thrombocytopenia, lactic acidosis, and hyponatremia [11–15]. Thus, therapeutic drug monitoring (TDM) of linezolid was strongly recommended, especially in the elderly [16]. Up to date, the proportion of persons aged 85 and over, the so-called “oldest old” is increasing dramatically worldwide [17]. However, there have been limited experiences of linezolid in the oldest old. Whether there was a relationship between the high mortality and linezolid overexposure was also not clear in patients aged 85 and over. Thus, we described the results of three years’ linezolid trough concentration and dose adjustment in elderly patients under the guidance of TDM and its effect on prognosis.

## Materials and methods

This retrospective study was conducted in ICU of Nanjing Drum Tower Hospital from January 2020 to November 2022. It involved 556 samples from 330 patients who received parenteral linezolid treatment with actual initial dosage of 600 mg twice daily longer than three days. Exclusion criterion was duration of linezolid therapy less than three days. This study was approved by the Medical Ethics Committee of the Nanjing Drum Tower Hospital, the Affiliated Hospital of Nanjing University Medical School (No. 2021–048-01). All methods were carried out in accordance with relevant guidelines and regulations that is declaration of Helsinki.

Patients were divided into three groups according to the age: aged < 65 years (young and middle aged), 65–85 years(the old), and ≥ 85 years(the oldest old). The first TDM was carried out ten minutes before the seventh dose, and the clinician adjusted the dose according to the first TDM and the patient's condition. As this is a retrospective study, there is no unified adjustment strategy. In general, for patients with linezolid C_min_ higer than 3 times the upper limit, clinicians usually chose drug withdrawal. For patients with C_min_ 1.5 to 3 times higher than the upper limit, the reduction scheme would be selected(400 mg, q12h). If the concentration was above the upper limit and within 1.5 times, usually no adjustment would be made. For patients’ linezolid C_min_ failed to reach 2 μg/mL, the dosage increase adjustment is 800 mg twice daily. The second TDM time varies from 2 to 5 days after adjustment, depending on the clinical decisions. Definition: Thrombocytopenia or neutropenia was defined as a 25% reduction from the baseline value.

### Statistical analysis

Statistical analyses were carried out by SPSS 21.0 software. Continuous variables with normal distribution were expressed as means ± standard deviations and were compared using the Student’s t-test, while continuous variables with non-normal distribution were presented as medians with their interquartile ranges (IQRs) and were compared using the Mann–Whitney U test. All categorical variables are presented as counts and percentages and were evaluated by the χ2 and Fisher's test. Correlations between the linezolid C_min_ and thrombocytopenia or neutropenia were analyzed by the Spearman’s rank test. Multivariate analyze was conducted to evaluate the factors for linezolid overexposure. Additionally, a binary logistic regression model was used to identify independent mortality factors in the oldest old. *P* < 0.05 was regarded as statistically significant.

## Results

Overall, data of 556 samples from 330 patients were collected from January 2020 to November 2022. All the linezolid C_min_ were considered for the statistical analyses.

### Distribution of linezolid C_min_ for the first TDM

Treated with the conventional dose of linezolid 600 mg twice daily, risks of overexposure to the drug were 75%, 54.8%, and 31.6% in patients aged ≥ 85, 65–84 and < 65 years respectively. There was no inadequate concentration in patients aged ≥ 85 years. Meanwhile, inadequate concentration existed in patients < 65 years with a risk of 24.7% (Table 1).

**Table 1:** The distribution of linezolid trough concentrations according in different groups

	≥ 85 years *n* = 32	65–84 years *n* = 124	< 65 years *n* = 174	*P*
> 7(μg/ml)	24 (75.0%)	69 (55.6%)	55 (31.6%)	< 0.001
2–7(μg/ml)	8 (25.0%)	42 (33. 9%)	76 (43.7%)	
< 2(μg/ml)	0 (0.0%)	13 (10.5%)	43 (24.7%)	

The proportions of the three groups who underwent continuous renal replacement therapy (CRRT)/hemodialysis (HD) are 30.3% (≥ 85 years), 33.1% (65–84 years), 38.5% (< 65 years) respectively, with no statistical differences

### The linezolid dosage adjustment according to TDM

The first TDM assessment led to the dose adjustment of 31.0%, 40.3%, 68.8% respectively in different groups (Table 2).

**Table 2:** The linezolid dosage adjustment according to the first therapeutic drug monitoring

	Withdrawal	Reduction	Replacement	Maintenance	Increased	Adjust in all
≥ 85 years (*n* = 32)	11(34.4%)	11(34.4%)	0(0.0%)	10(31.3%)	0(0.0%)	22 (68.8%)
65–84 years(*n* = 124)	21(16.9%)	23(18.5%)	5(2.2%)	74(59.7%)	1(0.8%)	50 (40.3%)
< 65 years (*n* = 174)	29(16.7)	12(6.9%)	5(2.9%)	120(69.0%)	8(4.6%)	54 (31.0%)

**Fig. 1:**
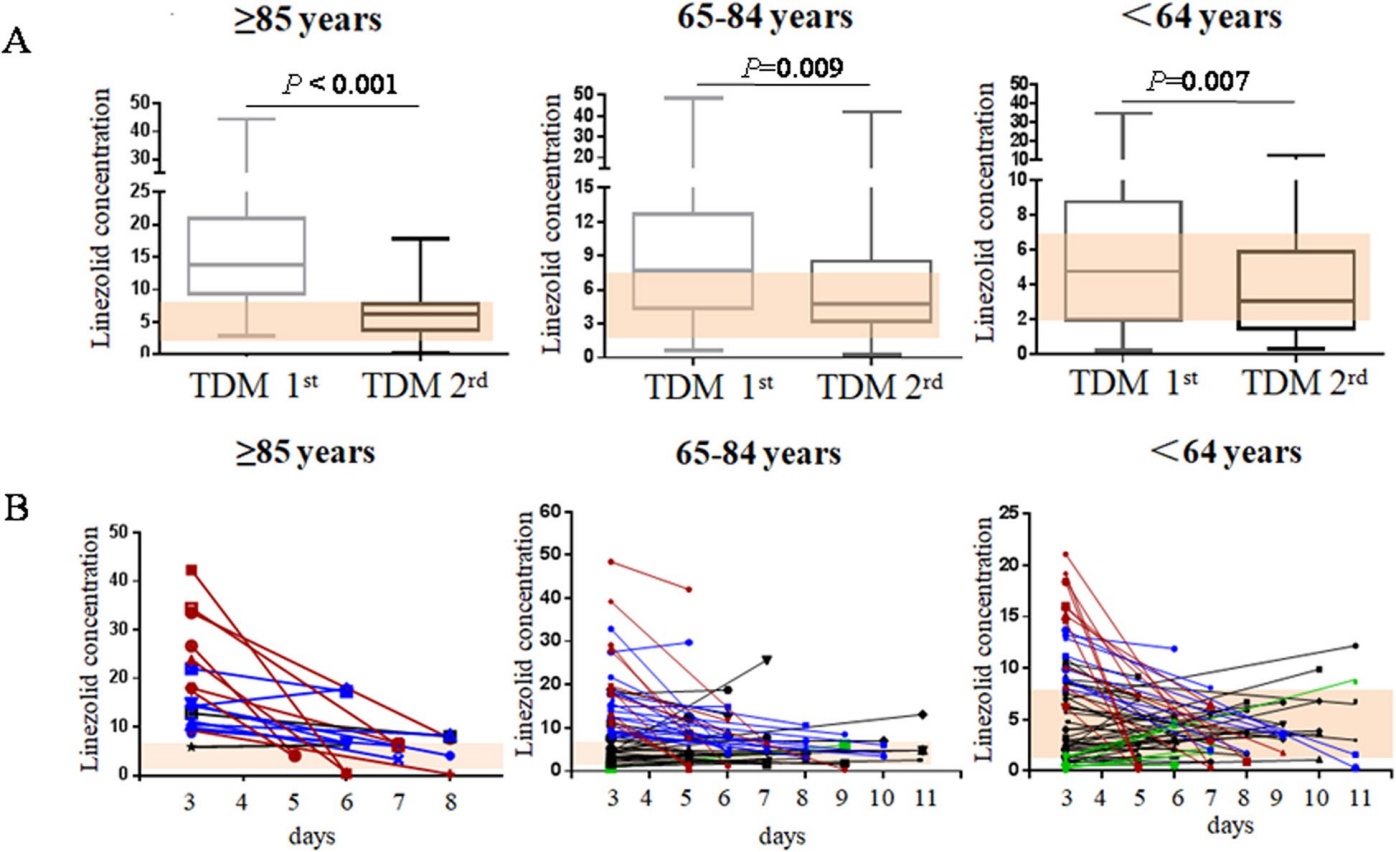
Linezolid plasma trough concentrations according to patients’ age. **A** Box-plot distribution of linezolid plasma trough concentrations clustered according to patients’ age. Horizontal solid lines represent the 5th, 25th, 50th, 75th, and 95th percentiles. The yellow area represents the therapeutic range for linezolid trough concentrations. **B** Line chart further revealed the linezolid adjustment according to patients’ age. The red lines indicate drug withdrawal. The blue lines indicate dosage reduction. The black lines indicate dosage maintenance. The green lines indicate dosage increased

The dose adjustment was determined according to the first TMD and the next TDM was conducted as required. As shown in Fig. 1A, linezolid dose adjustment helped more TDMs reach the proper C_min_ of 2–7 μg/ml, specifically, the proportion was 25.0% vs 66.7% in patients aged ≥ 85 years old, 34.7% vs 49.1% in patients aged 65–84 years old, and 43.7% vs 55.6% in patients aged < 65 years respectively. Line chart further revealed the linezolid adjustment according to patients’ age, as shown in Fig. 1B.

### The comparison between the old and the oldest old

Compared with the old group, the oldest old group linezolid C_min_ was nearly twice as high as that in the elderly group. There were also statistical differences between the two groups in urinary creatinine and creatinine clearance rate (Table 3), indicating that kidney function decreased with age, which may contribute to the overexposure of linezolid.

**Table 3:** The comparison between the old and the oldest old

Laboratory data	65–84 years old *n* = 156	≥ 85 years old *n* = 32	t/z	P
Linezolid C_min_ (μg/mL)	7.55 (4.32, 12.65)	13.60(7.28, 21.00)	-3.393	0.001
Alanine amino transferase (U/L)	20.40 (11.03,43.18)	21.40 (11.25,31.68)	-0.318	0.750
Aspartate aminotransferase (U/L)	28.80 (18.18,48.75)	33.65 (22.38,40.78)	-0.882	0.378
Total bilirubin (μmol/L)	13.35 (9.33,23.33)	10.95 (7.83,19.50)	-1.009	0.313
Albumin(g/L)	32.01 ± 4.43	32.51 ± 6.53	-0.516	0.607
γ-glutamyl transpeptidase (U/L)	36.20 (28.90,34.88)	36.45 (26.50,89.88)	-0.184	0.854
Lactate dehydrogenase (U/L)	290.00 (220.00,394.25)	270.00 (203.00,326.25)	-1.350	0.177
Choline esterase (U/L)	2.88 ± 1.32	2.61 ± 0.97	1.289	0.202
Blood urea nitrogen (mmol/L)	13.14 ± 8.34	12.43 ± 8.36	0.427	0.670
Serum Creatine (μmol/L)	86.50 (56.00,145.25)	72.50 (54.75,111.50)	-1.242	0.214
Creatinine clearance (ml/min)	52.62 (30.00,91.57)	35.36 (19.64, 61.79)	-2.251	0.024
Heamoglobin(g/L)	99.88 ± 22.23	93.00 ± 21.90	1.599	0.112
Platelets (× 10^9^/L)	170.04 ± 99.15	165.34 ± 82.81	0.251	0.802
Urine creatinine (mmol/12 h)	3095.66 ± 1988.56	2189.09 ± 1175.87	3.308	0.001

Linezolid C_min_, linezolid trough concentrations

### Risks for linezolid overexposure in patients over 65 years old

Multiple linear regression showed that creatinine clearance had negative relationship with linezolid concentration (*P* = 0.014) while the age was positive related to linezolid concentration (*P* = 0.003). Whereas, shock and body weight didn’t show correlation with linezolid concentration (Table 4).

**Table 4:** Risks for Linezolid overexposure in the elderly

	β	t	P
Shock or not	1.493	0.992	0.323
Creatinine clearance (ml/min)	-0.038	-2.495	0.014
Age(years old)	0.263	2.989	0.003
Body weight (kg)	-0.072	-0.878	0.323

### The correlation between linezolid overexposure and thrombocytopenia/ neutropenia

High concentration (> 10 μmol/mL) was associated with thrombocytopenia (rho = 0.165, *P* = 0.035), while it was not relative to neutropenia in the elderly. In contrast, there was no relationship between high linezolid concentration and thrombocytopenia in patients < 65 years (rho = 0.09, *P* = 0.240). The rate of thrombocytopenia in the three groups were 21.9%(≥ 85 years), 29.0%(65–84 years), 25.3%(< 65 years), with no statistical differences.

### The risks of the 28-day mortality in the oldest old

The mortality rate increased with age in critically ill patients. In order to explore whether high linezolid C_min_ increased mortality in the oldest old, we conducted the comparison between the survival and non-survivals. The results showed that there were statistical differences in norepinephrine maximum dosage (NE_max_), creatinine clearance, SOFA (sequential organ failure assessment), between the survival group and the non-survival group, while there was no statistical difference in high linezolid C_min_ (Table 5). Binary logistic regression showed that NE_max_ was an independent factor of 28-day mortality (Table 6).

**Table 5:** The comparison between the survivals and non-survivals in the oldest old

	Non-survivals(*n* = 11)	Survival (*n* = 21)	z/*χ*	*P*
Age (years old)	93.0 (88.0, 95.0)	87.0 ( 86.5, 91.0)	-1.860	0.063
Gender(male/%)	5 (45.5%)	5 (23.8%)	—	0.123
SOFA	11.00(6.00,12.00)	7.00(3.50,9.50)	-1.832	0.067
APACHE II	26.00(22.00, 35.00)	25.00(15.00, 31.00)	-1.113	0.266
Norepinephrine maximum dosage (μg/kg.min)	0.20(0.10, 0.54)	0.00(0.00, 0.06)	-3.531	0.001
Linezolid C_min_ (μg/mL)	14.30 (5.20, 28.40)	12.80(7.95, 20.00)	-0.377	0.706
Alanine amino transferase (U/L)	22.60 (4.80, 40.90)	20.80(11.60, 29.40)	-0.060	0.953
Aspartate aminotransferase (U/L)	35.50 (18.70, 62.90)	31.80(23.20, 35.65)	-0.694	0.092
Total bilirubin (μmol/L)	13.20 (9.90, 22.90)	9.00(6.85, 15.65)	-1.686	0.513
Albumin(g/L)	34.90 (32.60, 38.90)	32.20(29.85, 35.25)	-1.806	0.071
γ-glutamyl transpeptidase (U/L)	55.60 (23.60, 109.00)	33.40(27.20, 56.60)	-0.813	0.416
Lactate dehydrogenase (U/L)	275.00 (189.00, 323.5.00)	265.00(238.00,330.00)	-0.556	0.579
Choline esterase (U/L)	3.10 (1.20,3.40)	2.60(2.25, 3.25)	-0.099	0.921
Blood urea nitrogen (mmol/L)	15.30 (6.10, 21.30)	8.80(5.7, 13.25)	-1.290	0.197
Serum Creatine(μmol/L)	71.00 (60.00,126.00)	73.00(52.50,101.50)	-0.298	0.766
Creatinine clearance (ml/min)	22.18 (13.25, 29.58)	53.67(25.36, 68.53)	-2.400	0.016
Heamoglobin(g/L)	95.00(76.00, 116.00)	93.00( 80.00, 108.50)	-0.238	0.812
Platelets (× 10^9^/L)	172.00(87.00, 207.00)	172.00(125.00, 239.50)	-0.238	0.812
Urine creatinine (mmol/12 h)	1450.00(639.00, 2167.00)	2359.00( 1579.00,3599.00)	-2.162	0.031
Infection sites				
Pneumonia	7 (63.6%)	13 (61.9%)	—	1.000
Bacteremia	1 (9.1%)	2 (9.5%)	—	1.000
Abdominal infection	3 (27.3)	4 (19.0%)	—	0.667
Biliary tract infection	1 (9.1%)	1 (4.8%)	—	1.000
The others	2 (18.2)	1 (4.8%)	—	0.266
Causative organisms				
Gram-negative bacteria	9 (81.8%)	17 (81.0%)	—	1.000
Fungus	5 (45.5%)	13 (61.9%)	—	0.465

*SOFA* sequential organ failure assessment. *APACHE II* Acute Physiology and Chronic Health Evaluation II

**Table 6:** Binary logistic regression of risk factors for 28 day mortality in the oldest old

	β	SE	Wals	P	OR	95% CI	
						Lower	Upper
NE _max_	0.013	0.006	4.082	0.043	1.013	1.000	1.026

NE _max_ Norepinephrine maximum dosage, *SE* standard error, *OR* odds ratio, *CI* confidence interval

## Discussion

The proportion of the elderly in ICU with severe infection increased significantly. In this project, 30% of the patients are more than 65 years old. Due to the deterioration of organ function, the elderly are prone to drug accumulation and require dose adjustment [18]. However, experience with repeated monitoring of linezolid concentration and strategies in the elderly are limited. In this research, we evaluate linezolid trough concentration and dose adjustment in elderly patients under the guidance of TDM. Our analysis shows that the majority of the elderly treated with the in-label dose of linezolid (600 mg twice daily) had trough drug concentrations largely exceeding the upper safety threshold concentration, especially in those very old patients（≥85 years old）, even reached 42.4 μg/mL, six times fold over the upper safety concentration. Cattaneo D et al revealed the fact that nearly 50% and 70% of patients aged between 65 and 80 years and aged over 80 years, respectively, had linezolid trough concentrations over 8 mg/L [9]. In our study, the first TDM led to 68.8% dose adjustment in patients aged ≥85 years old and 40.3% dose adjustment in patients aged 65-84 years old respectively, indicating that overexposure had been the major problems in the elderly, and demanded for more attention. As the risk of adverse effects increases with the overexposure, TDMs are strongly recommended. Previously research showed that a decrease in linezolid dose to 300 mg twice daily after overexposure in the elderly ≥70 years old seemed proper, with 85% of the measurements falling within the therapeutic window [10]. In our study, the adjustment according to TDM mainly included drug withdrawal and dosage reduction (400mg, q12h) in the elderly. Linezolid dose adjustment helped the proportion of within the therapeutic window from 25.0% vs 66.7%, indicating that dose adjustment according to TDM benefits the elderly, especially the oldest old. 

The elderly inclined to linezolid accumulation may be related to progressive impairments in the functional reserve of multiple organs, resulting in an altered volume of distribution that might affect drug pharmacokinetics [19]. Renal dysfunction played an important role in the pharmacokinetics of linezolid [20]. It has been demonstrated that patients with renal impairment are more likely to experience accumulation of linezolid [21–23]. In patients with renal impairment, the median serum concentration of linezolid was 1.6-fold higher than in patients without renal impairment [21]. Crass RL et al. proved that age, body surface area, and estimated glomerular filtration rate were identified as covariates of linezolid clearance [20]. Abe S demonstrated that body weight BW and age were influential covariates on clearance of linezolid [8]. In this study, we also found that the oldest old was in high risk of linezolid overexposure with lower calculated creatinine clearance. Multivariate analyses linear regression revealed that age and CCR were related to linezolid concentration, while lower body weight was not. In addition, although volume of distribution in septic shock increased, Thallinger C found that there was no statistically significant difference in key pharmacokinetic parameters patients suffering from sepsis and septic shock [24]. Our research was consistent with previously study.

Insufficient linezolid C_min_ was also found in our study. Critically ill patients with augmented renal clearance, pediatrics, overweight, and obese patients are vulnerable to linezolid underexposure. It have been proved that continuous administration of linezolid might be critical for maximizing the time above the MIC (T > MIC) in those patients [25–27].

Overexposure increases the risks of adverse effect. As the literature has shown that the incidence of thrombocytopenia is more than 50% when the concentration of linezolid is higher than 10 μmol/L. Although the rate of thrombocytopenia in the three groups were with no statistical differences, We also proved that high concentration (> 10 μmol/L) was associated with thrombocytopenia in the elderly, indicating the necessity of TDM in the elderly.

It should be noted that critically ill patients usually receive co-treatments which may induce pancytopenia, such as antiviral drugs ganciclovir, acyclovir, ribavirin, oseltamivir, peramivir, etc., some antibiotics, β-lactamases, etc.; antifungal drugs, such as voriconazole, fluconazole, carpofungin, etc. The effect of co-treatment requires further investigation.

In addition, the mortality rate increased with age in critically ill patients. Exceeding the upper therapeutic safety threshold might lead to linezolid-related adverse events, whether might lead to high mortality was not sure. We found under the closely TDM and timely adjustment, the norepinephrine maximum dosage but not linezolid Cmin was associated with 28 day mortality in the oldest old, indicating that TMD was necessary to prevent undertreatment by inadequate low concentration and, and adverse effects by high concentrations cause in the elderly.

There are several limitations. First of all, this is a single center study with limited involving patients. Secondly, it is a retrospective study. Although all the first TDM of linezolid was determined, the second TDM was not assumed previously, with a span ranged from 2 to 5 days. This is not conducive to fully observe the concentration reduction rate and provide more convinced evidence for suggestion the TDM interval. In addition, the adjustment was determined by the doctor, not previously setting dosage, and failed to the influence of different adjustment schemes on concentration. At last, HD/CRRT might affect linezolid concentration because of the altered PK. However, as the time, duration, and therapeutic dose of hemofiltration were found to be different in retrospect among patients, they were not furthered explored.

## Conclusion

In conclusion, we conducted a study of three years’ linezolid adjustment experiences according to TDM in a real world and revealed the fact that TDM protected the elderly from the continuous exposure of linezolid. The high mortality of the oldest old was not related with linezolid overexposure under the closely TDM and timely adjustment.

Despite the limitations mentioned above, we believe it is important to afford more experiences on the use of linezolid in real-life practice. Besides, more studies are still needed to explore the proper initial dosage of linezolid.

## Data Availability

Alldatain the current study isavailable from the corresponding author on reasonable request.
